# Supplementary material for: Humanlike spontaneous motion coordination of robotic fingers through spatial multi-input spike signal multiplexing
Source: Nat Commun. 2023 Jan 3;14:5. doi: 10.1038/s41467-022-34324-3 (PMC9810717; doi:10.1038/s41467-022-34324-3)
Supplement: Supplementary file 1 — Supplementary Information [file 41467_2022_34324_MOESM1_ESM.pdf]

Supplementary information for

Humanlike Spontaneous Motion Coordination of Robotic Fingers Through Spatial Multi-input Spike Signal Multiplexing

Dong Gue Roe,[1,†] Dong Hae Ho,[2,†] Yoon Young Choi,[3] Young Jin Choi,[2] Seongchan Kim,[4] Sae Byeok Jo,[5] Moon Sung Kang,[6] Jong-Hyun Ahn,[1,] Jeong Ho Cho[2,*]

[1]School of Electrical and Electronic Engineering, Yonsei University, Seoul 03722, Republic of Korea.
[2]Department of Chemical and Biomolecular Engineering, Yonsei University, Seoul 120749, Republic of Korea.
[3]Department of Mechanical Science and Engineering, University of Illinois at Urbana−Champaign, Urbana, Illinois 61801, United States
[4]SKKU Advanced Institute of Nanotechnology (SAINT), Sungkyunkwan University, Suwon 16419, Republic of Korea.
[5]School of Chemical Engineering, Sungkyunkwan University, Suwon 16419, Republic of Korea.
[6]Department of Chemical and Biomolecular Engineering, Institute of Emergent Materials, Sogang University, Seoul 04107, Republic of Korea

*Corresponding author: J. H. C (jhcho94@yonsei.ac.kr)
[†]D. G. R and D. H. H contributed equally to this work.

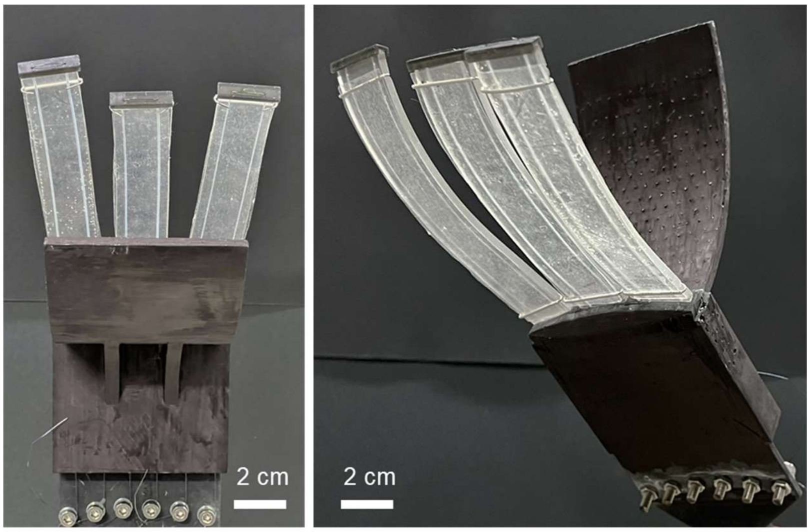

Fig. S1. Photographs of the CRH. Photographic image of CRH on different views.

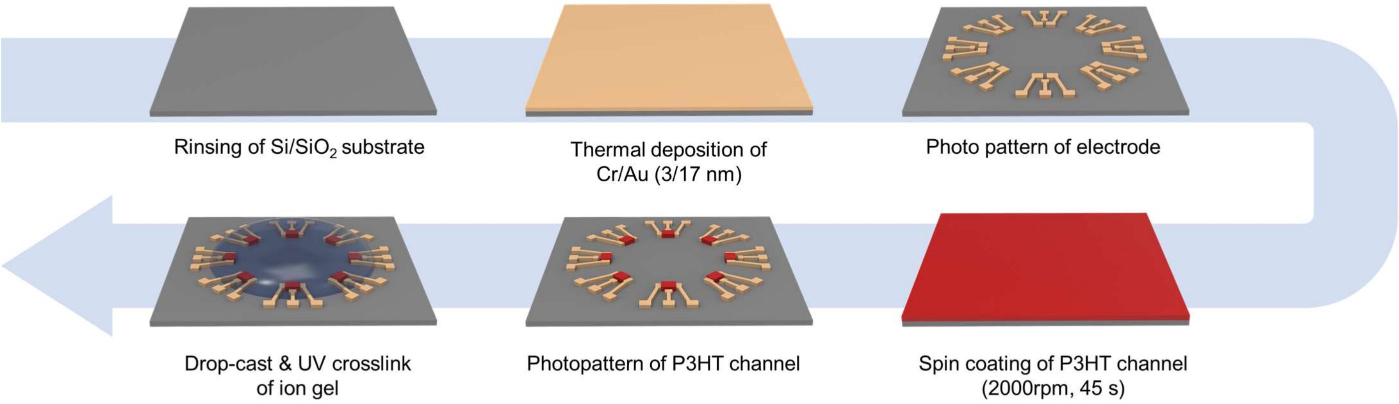

Fig. S2. Fabrication process for the PPSA. Fabrication process for the PPSA using conventional photolithography, spin-coating and drop-cast methods.

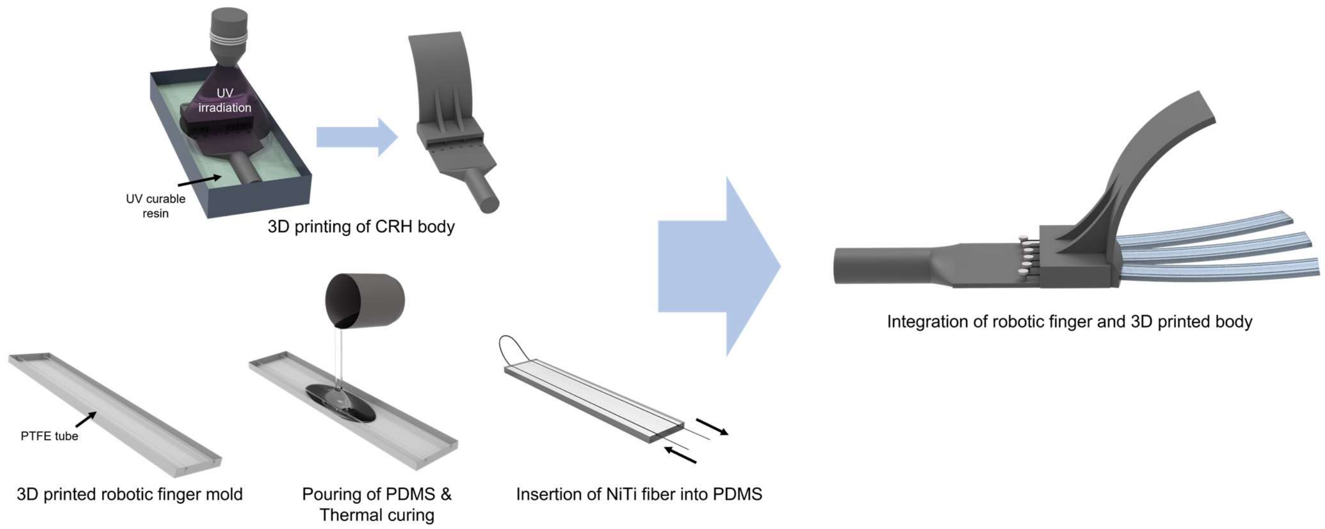

Fig. S3. Fabrication process for the CRH. Fabrication process for the CRH using 3D printing and thermal curing method of PDMS.

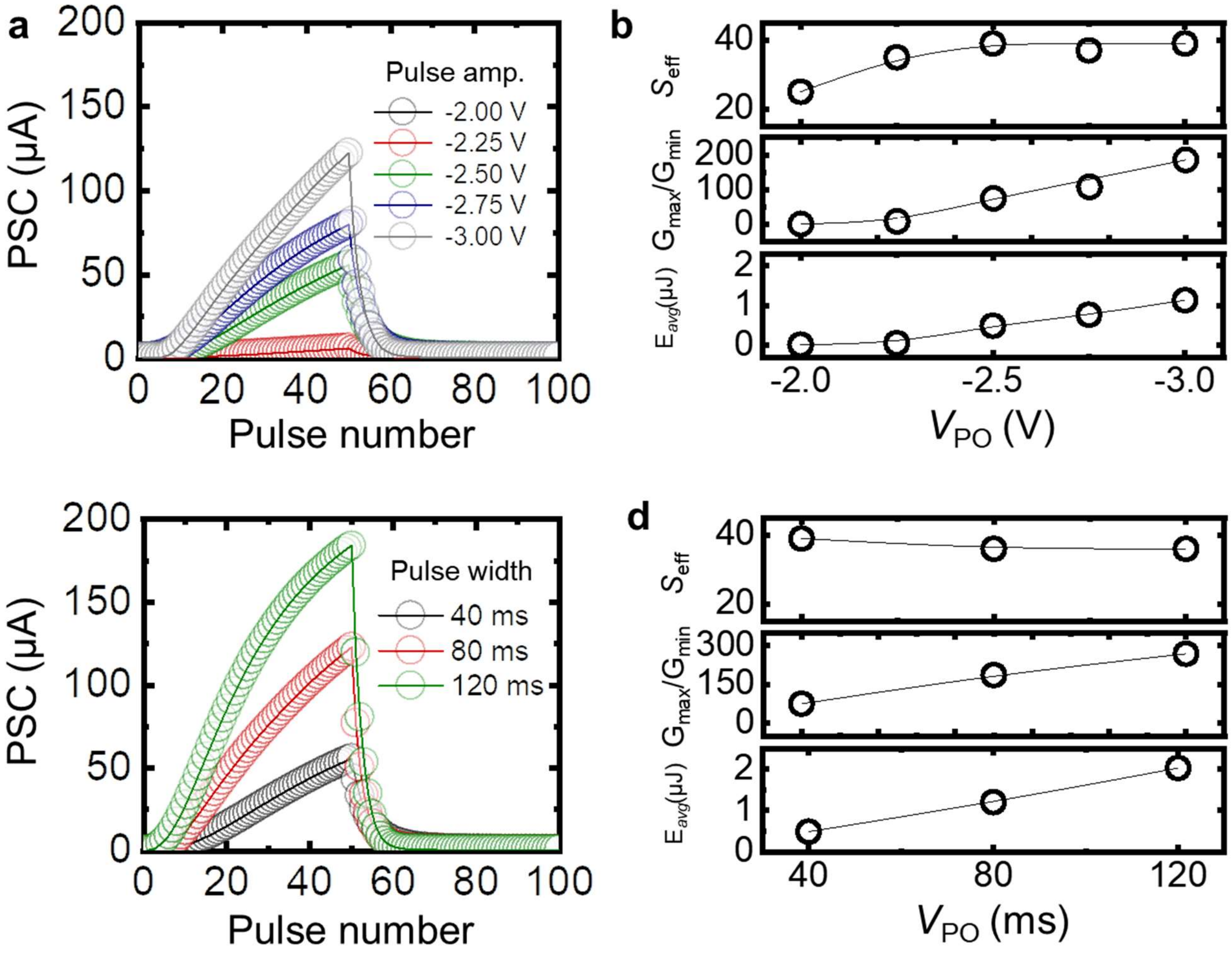

Fig. S4. Synaptic performance for various pulse conditions. a PSC change for different amplitudes of V_{PO} from -2.0 V to -3 V. **b** Synaptic properties for varying amplitude of V_{PO} from -2.0 V to -3 V. **c** PSC change for different pulse widths of V_{PO} from 40 ms to 120 ms. **d** Synaptic properties for varying amplitude of V_{PO} from 40 ms to 120 ms.

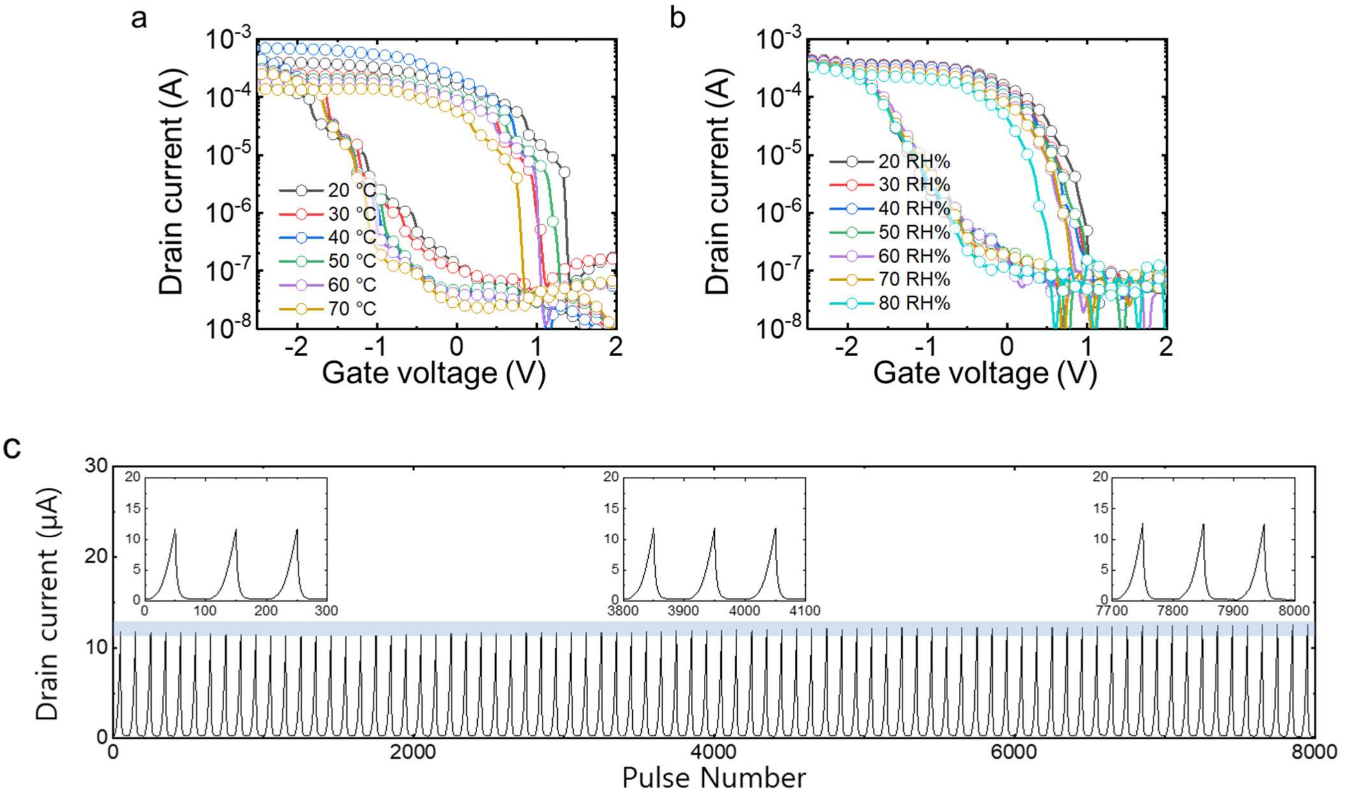

Fig. S5. Environmental stability and repeatability of OAST. a Transfer curves of OAST under varying temperature. **b** Transfer curves of OAST under varying humidity. **c** Repeatability of OAST under applying 8,000 LTP/D pulses.

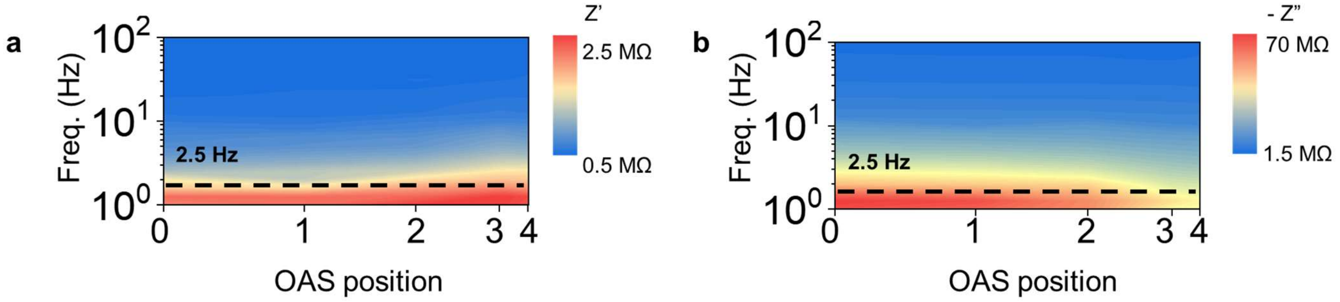

Fig. S6. Impedance properties for different transistor positions. a Z' change for different transistor positions. **b** $-Z''$ change for different transistor positions.

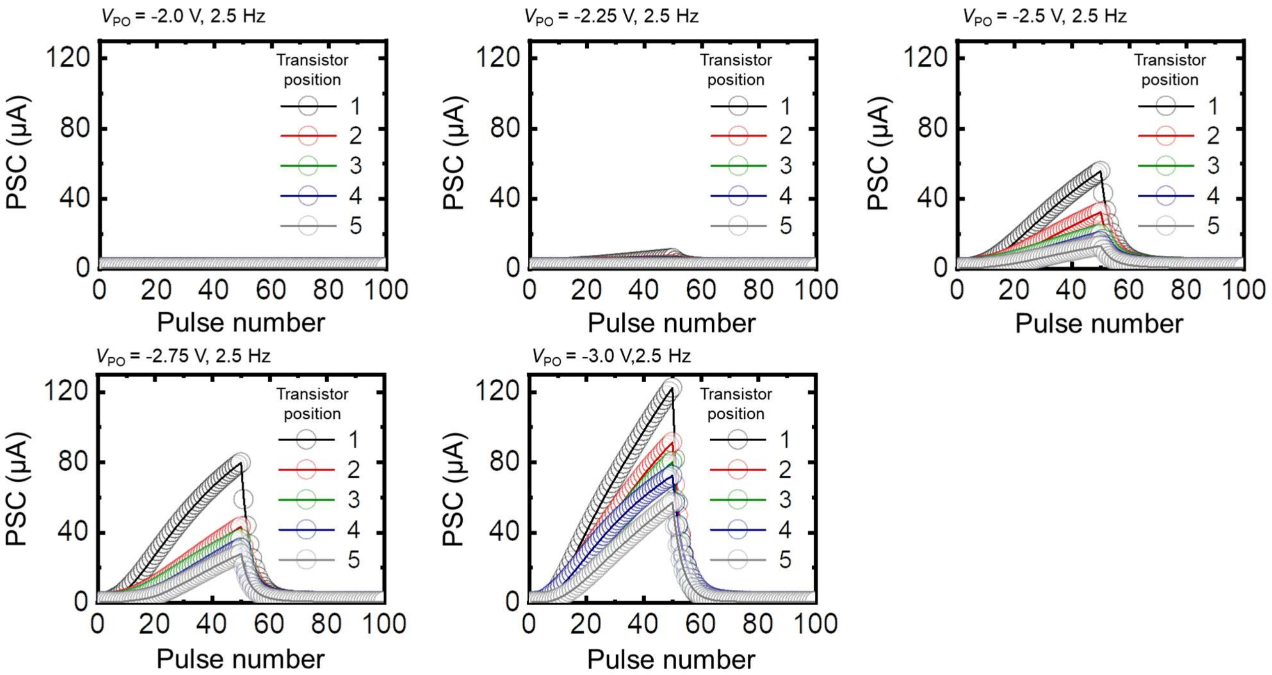

Fig. S7. PSC change for different OAST positions and for different V_{PO} amplitudes from -2.0 V to -3 V. The PSC was measured with varying position and voltage amplitude of V_{PO}. The largest difference in the PSC for inputs at Positions 0 and 4 was obtained using a 2.5 Hz V_{PO} pulse with a magnitude -2.5 V and a width of 40 ms.

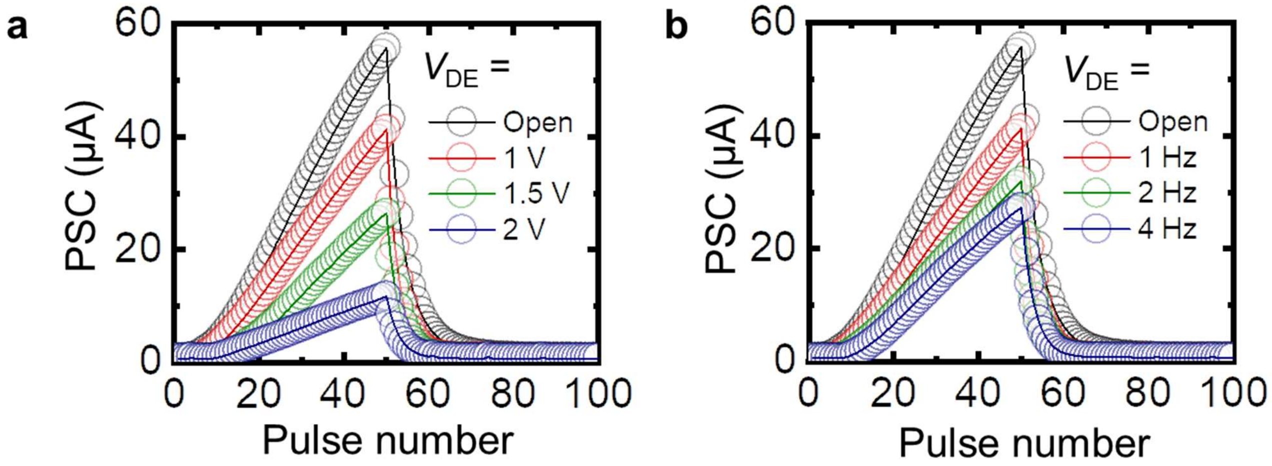

Fig. S8. PSC change for different amplitudes and frequencies of V_{DE} at position 1 when V_{PO} (-2.5 V, 2.5 Hz) was applied at position 0. a PSC change for different amplitudes of V_{DE} (open to 2 V). **b** PSC change for different frequencies of V_{DE} (open to 4 Hz).

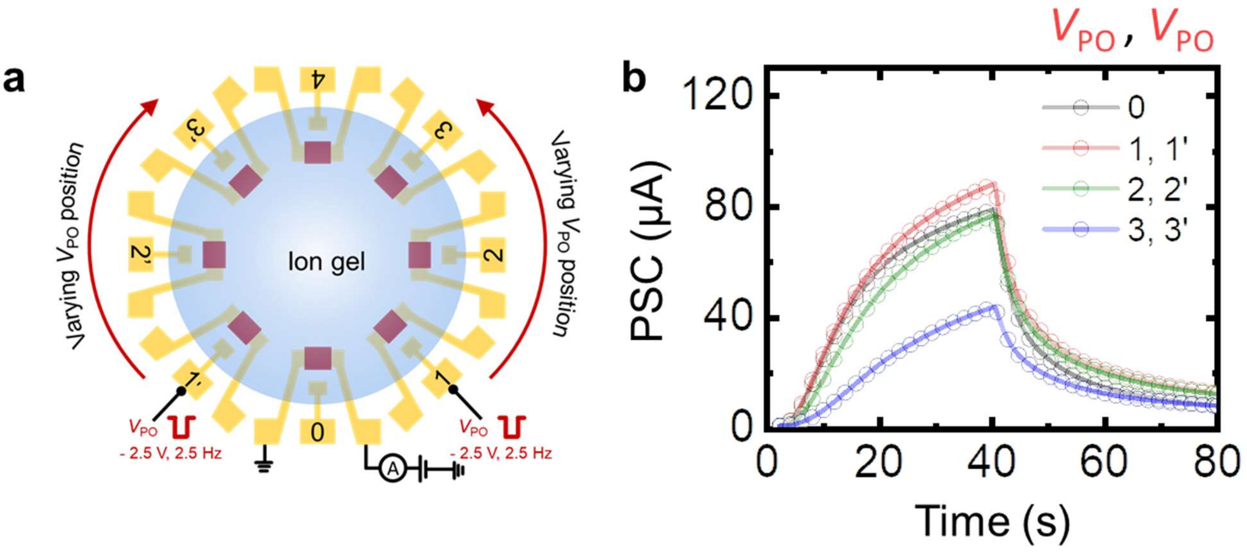

Fig. S9. PSC change when multiple V_{PO}'s were applied at different positions. a Schematic of the measurement conditions and **b** the PSC change for multiple V_{PO}'s and a single V_{PO}.

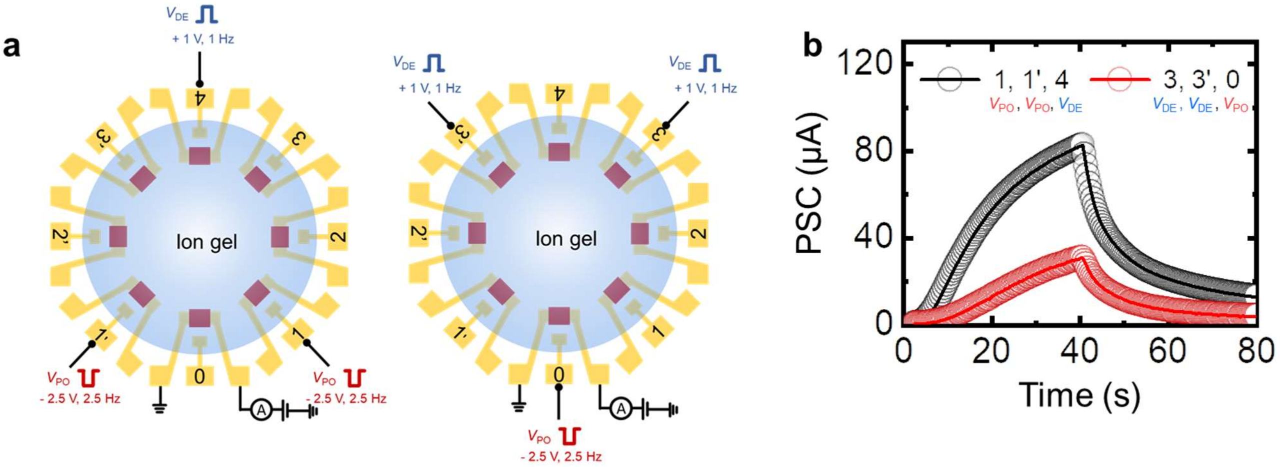

Fig. S10. PSC change for a combination of V_{PO}'s and V_{DE}'s. a Schematic of measurement conditions and **b** the PSC change for multiple V_{PO}-V_{DE} combinations (V_{PO}, V_{PO}, V_{DE} = 1,1′, 4 and V_{DE}, V_{DE}, V_{PO} = 3, 3′, 0).

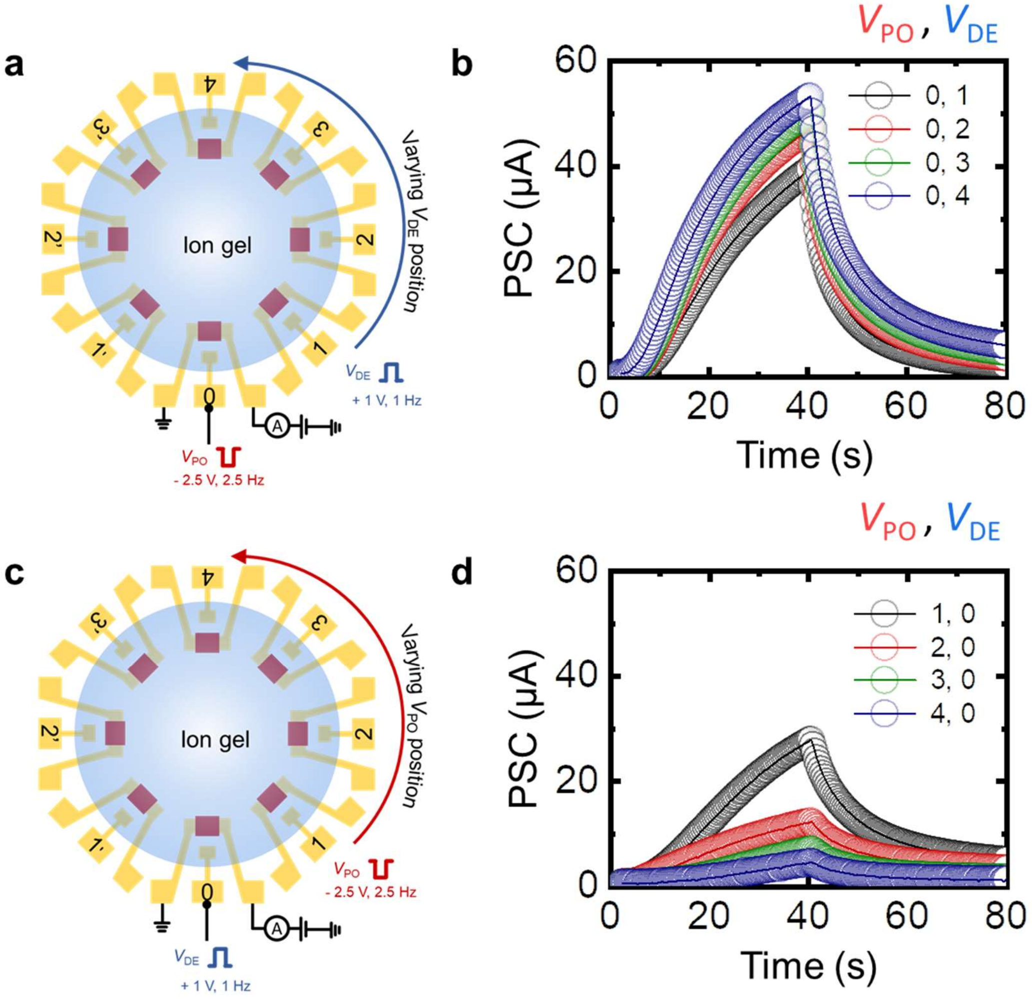

Fig. S11. PSC change for different positions of V_{PO} and V_{DE}. (A) Schematic of measurement conditions (V_{PO} position = 0 and V_{DE} position = 1 to 4). (B) PSC change for a fixed V_{PO} and a varying V_{DE}. (C) Schematic of measurement conditions (V_{DE} position = 0 and V_{PO} position = 1 to 4). (D) PSC change for a fixed V_{DE} and a varying V_{PO}.

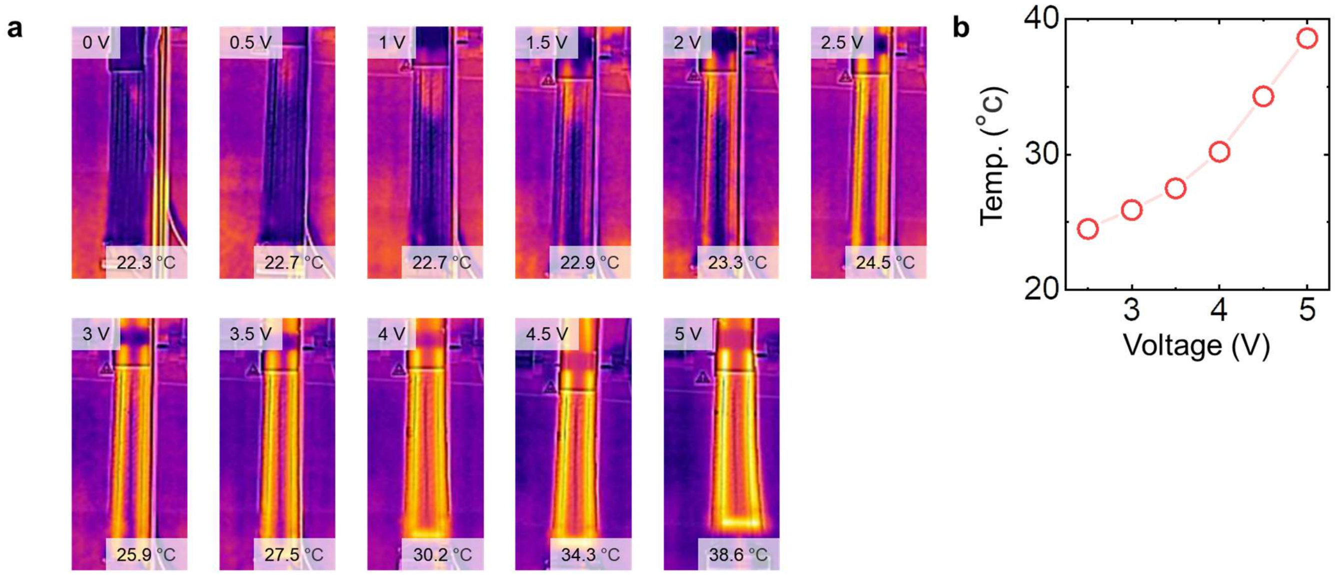

Fig. S12. Measured temperature of robotic finger for different applied voltages. a Thermal images of a robotic finger for different applied voltages (0 V to 5 V). **b** Measured temperature of a robotic finger for different applied voltages.

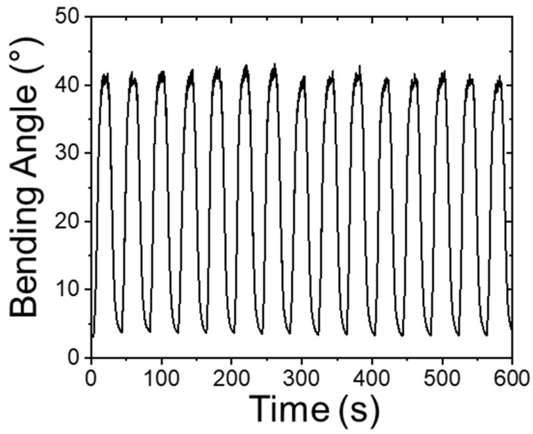

Fig. S13. Repeatability test results of the robotic finger. The repeatability of the robotic finger was tested by performing 15 consecutive actuation tests.

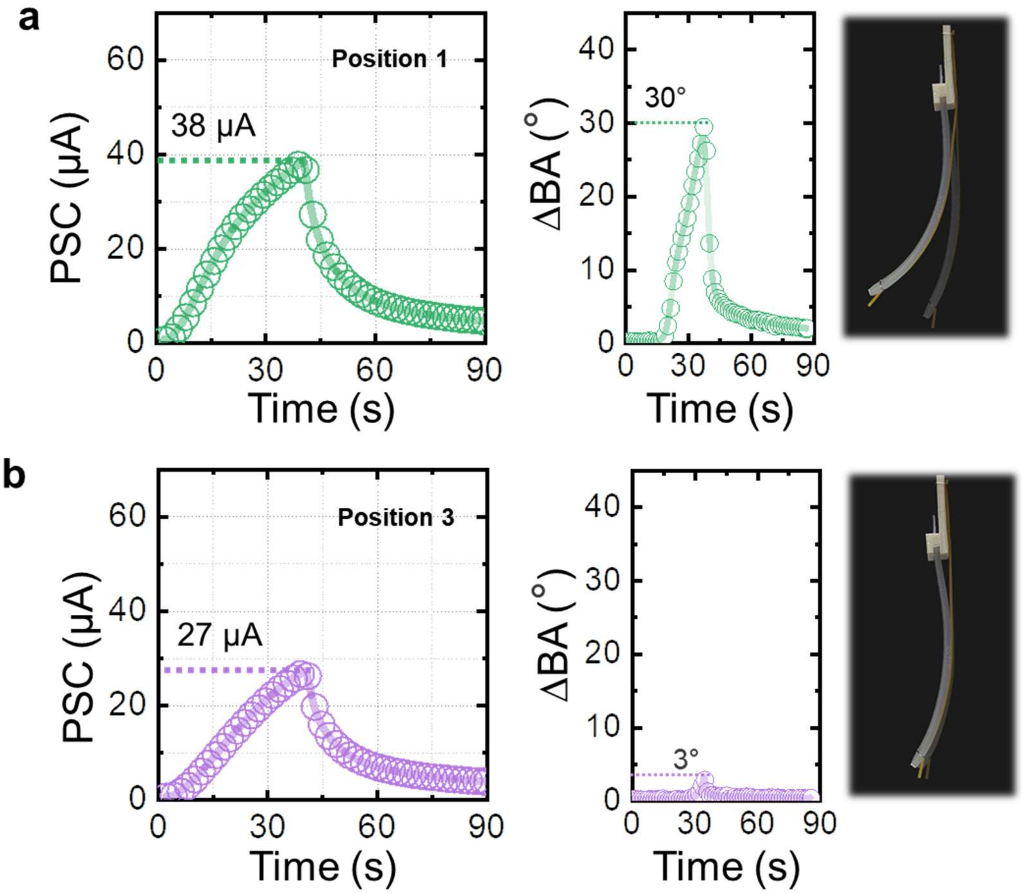

Fig. S14. Measured PSC and bending angle of a robotic finger for different OAST positions. a PSC and ΔBA of the robotic finger at OAST position 1. **b** PSC and ΔBA of the robotic finger at OAST position 3.
