# Supplementary material for: Humanlike spontaneous motion coordination of robotic fingers through spatial multi-input spike signal multiplexing
Source: Nat Commun. 2023 Jan 3;14:5. doi: 10.1038/s41467-022-34324-3 (PMC9810717; doi:10.1038/s41467-022-34324-3)
Supplement: Supplementary file 3 — Description of Additional Supplementary Files [file 41467_2022_34324_MOESM3_ESM.pdf]

Description of Additional Supplementary Files

File Name: Supplementary Video 1

Description: Video of cup grabbing motion by PPSA control system

File Name: Supplementary Video 2

Description: Video of cup grabbing motion by conventional control system
